# The Relative Contribution of Executive Functions and Aging on Attentional Control During Road Crossing

**DOI:** 10.3389/fpsyg.2022.912446

**Published:** 2022-05-12

**Authors:** Victoria I. Nicholls, Jan M. Wiener, Andrew Isaac Meso, Sebastien Miellet

**Affiliations:** ^1^Department of Psychology, University of Cambridge, Cambridge, United Kingdom; ^2^Ageing and Dementia Research Centre, Bournemouth University, Poole, United Kingdom; ^3^Neuroimaging Department, Institute of Psychiatry, Psychology and Neuroscience, King's College London, London, United Kingdom; ^4^School of Psychology, University of Wollongong, Wollongong, NSW, Australia

**Keywords:** visual attention, eye movements, scene perception, aging, pedestrian safety, executive functions

## Abstract

As we age, many physical, perceptual and cognitive abilities decline, which can critically impact our day-to-day lives. However, the decline of many abilities is concurrent; thus, it is challenging to disentangle the relative contributions of different abilities in the performance deterioration in realistic tasks, such as road crossing, with age. Research into road crossing has shown that aging and a decline in executive functioning (EFs) is associated with altered information sampling and less safe crossing decisions compared to younger adults. However, in these studies declines in age and EFs were confounded. Therefore, it is impossible to disentangle whether age-related declines in EFs impact on visual sampling and road-crossing performance, or whether visual exploration, and road-crossing performance, are impacted by aging independently of a decline in EFs. In this study, we recruited older adults with maintained EFs to isolate the impacts of aging independently of a decline EFs on road crossing abilities. We recorded eye movements of younger adults and older adults while they watched videos of road traffic and were asked to decide when they could cross the road. Overall, our results show that older adults with maintained EFs sample visual information and make similar road crossing decisions to younger adults. Our findings also reveal that both environmental constraints and EF abilities interact with aging to influence how the road-crossing task is performed. Our findings suggest that older pedestrians' safety, and independence in day-to-day life, can be improved through a limitation of scene complexity and a preservation of EF abilities.

## Introduction

As we age, many physical, perceptual, and cognitive abilities decline, and these declines can have a critical impact on our day-to-day lives. However, the decline of many abilities is concurrent; thus, it is often very challenging to disentangle the relative contributions of different abilities in the performance deterioration in complex, realistic tasks with age.

Road-crossing is a very interesting context to study the complex coordination of many abilities that are involved in real-life situations. Road crossing is highly socially relevant, it is a common yet challenging task, performed in most countries and by all age groups, involving body, head, and eye movements, moving targets, integration of information from different parts of the visual fields, and requires fast decision making with potentially dire consequences. Critically, road-crossing is an activity in which older adults are particularly vulnerable. Older adults (above 75y/o) have the highest rate of pedestrian accidents in Australia (BITRE, 2015), and in the EU older adults make up nearly half of all pedestrian fatalities (ERSO, 2018).

Age-related cognitive declines of visual attentional control and executive functioning (EFs) have been proposed to explain the vulnerability of older adults in complex day-to-day tasks such as road-crossing (Nagamatsu et al., 2011; Dommes et al., 2013; Geraghty et al., 2016). Visual attentional control includes abilities such as suppressing task-irrelevant distractors (Milham et al., 2002) or switching between targets (Hampshire et al., 2008), while EF includes abilities such as inhibition, planning, working memory, and cognitive flexibility (Anders et al., 1972; Olincy et al., 1997; Butler et al., 1999; Milham et al., 2002; Allain et al., 2005; Hampshire et al., 2008; Beurskens and Bock, 2012).

The decline of these cognitive abilities has been shown to impact tasks related to road-crossing, such as walking or driving. In walking, older adults need more time to process visual information and plan accurate stepping movements (Chapman and Hollands, 2006, 2007; Zietz and Hollands, 2009). For driving, studies have linked decline in attentional control in older adults to difficulties in vehicle navigation (Romoser et al., 2013) and motor vehicle crash rates (Shinar et al., 1978), and decline in working memory and attention to problems in hazard detection during driving (Ponds et al., 1988; Plude and Doussard-Roosevelt, 1989; Stine and Wingfield, 1990; Parasuraman and Nestor, 1991; Caird and Chugh, 1997; Kidder et al., 1997; Fisk and Warr, 1998; Ho et al., 2001; Horswill et al., 2008).

Regarding road crossing, a task that requires walking, navigation, and hazard detection, studies on older adults have linked deficits in attention switching (Dommes et al., 2013), and spatial planning (Geraghty et al., 2016) to fewer safe crossing decisions. The potential involvement of attentional control and EFs in older pedestrian safety led (Zito et al., 2015) to investigate the relationships between aging, EFs, visual sampling, and road crossing decisions. They found that older adults spent more time than younger adults looking at the ground in front of them. Moreover, older adults made more unsafe crossing decisions and showed declines in EFs. However, in three studies investigating visual exploration in age-related decline of road crossing performance, age, and EFs decline were confounded (Dommes et al., 2013; Zito et al., 2015; Geraghty et al., 2016). Therefore, it is impossible to disentangle two potential scenarios: on the one hand, the age-related declines in EFs might impact on oculomotor inhibition and switching abilities, thus affecting visual sampling and in turn road-crossing performance. On the other hand, it is possible that visual exploration and road-crossing performance are impacted by aging independently of a decline in EFs (e.g., by perceptual, cognitive, and/or motor slowness).

Although cognitive decline occurs commonly with age (Hedden and Gabrieli, 2004), some research suggests that it is not inevitable, leading to the concept of “SuperAgers” (Rogalski et al., 2013; Gefen et al., 2015). Super agers are typically defined as older adults above the age of 80 that show cognitive abilities comparable to middle-aged individuals (Harrison et al., 2012; Gefen et al., 2014; Huentelman et al., 2018; Rogalski, 2019; Yang et al., 2019; De Godoy et al., 2021). Yang et al. (2019) also suggests that the EF abilities of super agers would be above that of normally aging older adults. Even below the age of 80, some older adults show similar characteristics to super-agers, i.e., similar EF abilities compared to young adults. Thus, these individuals might be on the trajectory to become super-agers. In the present study, we recruited such older adults which allowed us to differentiate the impacts of aging from the impacts of a decline in EFs. This allowed us to directly address the following question: Does aging have an impact on visual attentional control and road crossing abilities independently of a decline in EFs? Alternatively, do aging and EF abilities interact in day-to-day activities such as road-crossing?

We presented younger adults and older adults with videos of road traffic and asked them when they could cross the road while we recorded their eye-movements. We included varying levels of traffic density, thus differential cognitive and perceptual loads, which could influence attentional shift. Additionally, we included pedestrians, which allowed us to assess inhibitory control as it is known that people induce attentional capture (Birmingham et al., 2008; Foulsham et al., 2010).

## Methods

### Participants

Sixty-four participants were recruited, 31 older adults aged between 60 and 83 years old (y/o, mean = 69.03, SE = 1.38), and 33 younger adults aged between 18 and 35 y/o (mean = 22.37, SE = 0.91). All younger adults were recruited at Bournemouth University, UK. We recruited older adults from the Bournemouth University Aging and Dementia Research Centre participant pool (specifically older adults without identified cognitive deficit) and from the Bournemouth branch of the University of the Third Age. Participants from these groups are typically very physically, and socially active which helps to maintain high-level of EF abilities as individuals age (Kramer et al., 1999; Derwinger et al., 2005; Carlson et al., 2008; Ybarra et al., 2008; Berryman et al., 2013).

All participants had normal or corrected to normal vision. Participants were also screened for mild cognitive impairments using the MoCA (Nasreddine et al., 2005). One older adult was excluded based on a cut-off score of 23 (Luis et al., 2009). An additional older adult and three younger adults were excluded for poor eye tracking recording. We define poor recording as tracking loss for more than 50% of the data. Therefore, 29 older adults and 30 younger adults were included in the final analyses. The study was approved by Bournemouth University's ethics committee. Informed consent was obtained from participants prior to taking part. Participants took part in exchange for course credits or monetary compensation at a rate of £10/h for their time. This study was performed in accordance with all appropriate institutional and international guidelines and regulations, in line with the principles of the Helsinki Declaration.

### Executive Function Tests

To confirm that the older participants had maintained EF abilities we used the BADS zoo map test (Wilson et al., 1996), and the Rogers and Monsell (1995) attention shift paradigm (RMA).

The BADS zoo map test measured the participants' spatial planning ability by assessing participants' ability to plan a route around a zoo. Participants were scored based on visiting the correct locations and points were deducted when participants broke the rules and exceeded time limits for planning.

The RMA assessed participants' attentional control by getting participants to switch between two similar tasks. For the RMA task, we extracted the global and local switch costs as done by Rogers and Monsell (1995). The global switch costs refer to the difference in performance between a block where participants perform the same task and a block where participants are switching between tasks. Local switch costs refer to the differences in performance between switch and non-switch trials.

Both tests have previously been linked to road crossing ability (Dommes et al., 2013; Geraghty et al., 2016) and were designed to assess participants' spatial planning and attention shifting abilities.

### Apparatus

During the experiment participants' eye movements were recorded at a sampling rate of 1,000 Hz with the SR-Research EyeLink 1000 (with a chin and forehead rest), which has an average gaze position error of about 0.25° and a spatial resolution of 0.01°. Only the dominant eye was tracked. Stimuli were presented on an HP monitor with a screen resolution of 1,920 by 1,080 pixels, a width of 534 mm and a height of 300 mm, a horizontal viewing angle of 46.9° and a vertical viewing angle of 27.4° at a distance of 740 mm. The experiment was coded in Matlab (MATLAB, 2016) using the Psychophysics toolbox, PTB-3 (Brainard, 1997) and EyeLink Toolbox extensions (Cornelissen et al., 2002). Calibrations for eye fixations were conducted at the beginning of the experiment using a nine-point fixation procedure as implemented in the EyeLink API (see EyeLink Manual) and using Matlab software. Calibrations were then validated with EyeLink software and repeated until there was <1° of error for every calibration point.

### Experimental Procedure

At the start of the experiment participants would take the executive functioning tests, which lasted for ~30 min. After these tests participants would complete the road crossing task.

**Figure 1:**
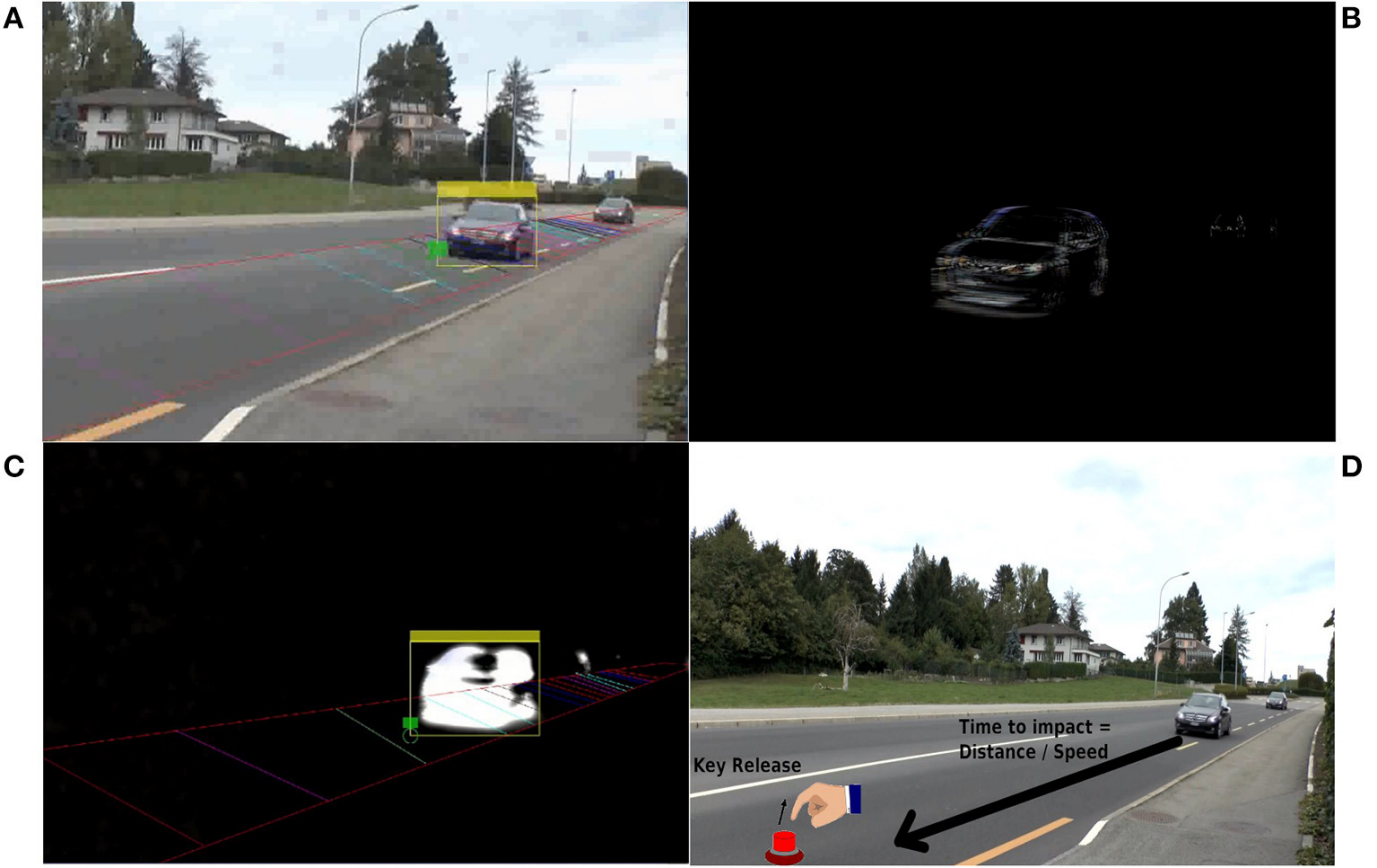
Illustration and description of the car detection algorithm. **(A)** Screenshot of the car detection algorithm on original stimuli. Colored markers on the road indicate where car distance is calculated. **(B)** Difference video. **(C)** Difference video features magnified by the Eulerian magnification with results of the car detection algorithm. **(D)** Illustration of the time to impact measure. Description of algorithm: Our method uses a foreground detector *via* Gaussian mixture models (Kingdom, 2017) then performs a Blob Analysis on the detected foreground objects. We then applied a Kalman filter (Kingdom, 2019) to reduce the number of times the objects were lost **(A)**. To further improve the performance of the foreground detector we created difference videos from the stimuli videos. In the difference videos, each frame was created by subtracting the previous frame in the original video from the current one **(B)**. Moreover, the motion in each difference video was enhanced using the Eulerian magnification toolbox (Wu et al., 2012). We amplified the motion so that the vehicles blurred into one very bright object—including larger vehicles (such as trucks) which would often be detected as two objects by the car detection algorithm **(C)**. A marker was then placed in the video at known distances along the road and the time at which the car passed over these markers was calculated **(A)**.

For the road crossing task we used the same video stimulus and design as in Nicholls et al. (2019). At the beginning of the task participants were informed that they would be presented with a series of videos of road crossing situations and that they would have to indicate by pressing the spacebar on a keyboard when they could cross the road and hold the button down for as long as they thought it was safe to cross. Participants were instructed to focus on vehicles on the side of the road closest to them. Vehicles traveled at an average velocity of 50 km/h. Each trial started with the presentation of a central fixation cross. Once the participants had fixated on the cross a blank screen was presented for 500 ms and then the video clip for the trial was presented (see Figure 1A). Each trial was followed by another blank screen for 500 ms, and the next trial started with the central cross. One hundred trials were presented to the participants each with a different video clip, each lasting 10 s, giving a total task time of ~30 min and a total experiment time of 1 h. The number and duration of button presses for each trial were collected and analyzed. The videos were completely natural, and no aspects of the videos were staged, and they were not edited to control when the cars emerged. Thirty-five of the videos contained pedestrians. As our stimuli were natural videos, they include a variety of pedestrians of various ages, ethnicities, body shapes, clothing styles and colors, carrying objects or not, some with pets and so on. Therefore, the pedestrians in our videos are expected to have a variety of saliencies reflecting the real world. The quality of the videos does not allow the pedestrians to be identified and the videos were approved by the ethics committee at the University of Fribourg. The camera was always fixed in the same location, at a height in between the average adult and the average child's height. The video clips did not include sound.

As the video clips in Nicholls et al. (2019) were filmed at a real road crossing in Fribourg the driving direction would be incorrect for participants in the UK. Therefore, the videos were mirrored to simulate a road crossing in the UK. Critically, registration numbers were not identifiable, and the visual scenes did not include any information allowing participants to identify where they were filmed. Prior to the experiment, 10 British drivers were asked where the video clips were located, all of whom responded with a location in the UK.

### Statistical Analysis

All statistical analyses and figures were performed and created using Matlab 2016a (MATLAB, 2016) and R (R Core Team, 2016).

#### Crossing Decisions

The number and duration of crossing decisions were analyzed with linear mixed models (LMMs) using the lme4 package in R (Bates et al., 2015). To get an indication of how much time participants gave themselves to cross the road, we also calculated the time to impact (TTI). We defined “time to impact” as the time that it would take for the closest approaching vehicle to reach the participants, from the moment when the participants stopped indicating that crossing was safe (i.e., when they released the response button indicating that it was no longer safe to cross). This is illustrated in Figure 1D.

Each of the three models had fixed effects of age group, BADS zoo map score, local switch cost, global switch cost, traffic density, and distractors. Each of the models included 11 interactions.

We selected the interactions to directly assess our research questions. We wanted to know if our experimental manipulations (distractors and traffic density) have differential effects for younger and older adults. We also wanted to assess if age groups interact with our measures of executive functioning or, alternatively, if age, and executive functioning (as measured here) have independent effects on crossing decisions. Finally, we wanted to verify that our experimental manipulations, tapping into inhibition and attention switching, interact with our measures of executive functioning.

The model for time to impact and number of crossing decisions also included random intercepts for each participant and video and random slopes for zoo map score. The model of button press duration only included random intercepts for each participant and each video. The random effects structure initially included slopes for each fixed factor and interaction, but this model did not converge, and so the random effects structure was pruned using the procedure described by Bates et al. (2015). The full model for time to impact and number of crossing decisions is summarized in the below formula:


$$\begin{array}{r} \;DV(number\;of\;button\;presses/TTI)\;\sim \;Age\;group\;*\;(distractors\\ +\;traffic\;density)+BADS\;zoo\;map\;score\;*\;(Age\;group+distractors\\ \;+\;traffic\;density)+local\;switch\;cost\;*\;(Age\;group+distractors\\ \;+\;traffic\;density)+global\;switch\;cost\;*\;(Age\;group+distractors\\ \;+\;traffic\;density)+(1+BADS\;zoo\;map\;score|participant)\\ \;+\;(1+BADS\;zoo\;map\;score\;|\;video\;clip) \end{array}$$

The full model for the duration of crossing decisions can be summarized in the below formula:


$$\begin{array}{r} \;Duration\;\sim \;Age\;group\;*\;(distractors+traffic\;density)\\ +\;BADS\;zoo\;map\;score\;*\;(Age\;group+distractors+traffic\;density)\\ \;+\;local\;switch\;cost\;*\;(Age\;group+distractors+traffic\;density)\\ \;+\;global\;switch\;cost\;*\;(Age\;group+distractors+traffic\;density)\\ \;+\;(1|participant)+(1|video\;clip) \end{array}$$

For each of the models the global and local switch cost measures were log transformed as they were not normally distributed.

For each video clip, the presence of a human distractor was encoded in a dichotomous way (1 for one or more human distractors present in the trial, 0 for no human distractors in the trial). The number of vehicles and vehicle locations at each video frame were determined using a custom automatic car detection Matlab algorithm (see Figure 1). From the automatic car detection, the location and time of the vehicles were used to calculate how long it would take the vehicles to reach the participants from the time the participant released the response button indicating a safe crossing, i.e., the time to impact. A large time to impact value would indicate an early and safe decision.

#### Executive Function Tests

Differences between older adults and younger adults on all measures were determined using a bootstrap *t*-test with a one-step M-estimator with the rogme package in R (Rousselet et al., 2017). Multiple comparisons were corrected using the Hochberg method. We used bootstrap *t*-tests as they better take into account the true data distributions compared to parametric tests based on theoretical distributions (Rousselet et al., 2019). Bayes factors were also calculated using the BayesFactor package in R (Morey and Rouder, 2018), after outliers were removed.

#### Statistical Analysis of Eye Movements

Eye movements were parsed into fixations, saccades and smooth pursuits using the same custom algorithm as in Nicholls et al. (2019; Supplementary Figure 2).

Gaze samples were analyzed using statistical gaze maps. Statistical maps were calculated using iMap4 (Lao et al., 2017). iMap4 computes pixel-wise LMMs across participants and trials on each gaze map and uses a bootstrap cluster correction for multiple comparisons.

The linear mixed model used for iMap4 had the same fixed and random effects structure as the model used with the duration of crossing decisions data described above. The aim in using iMap4 was to determine where participants looked during the videos depending on scene characteristic such as the presence of pedestrians or the traffic density, and participants characteristics such as age group or EF scores.

## Results

### Executive Function Tests

We confirmed that the older adults in this study had maintained EFs. Indeed, they performed in the same range as younger adults for all EF measures (Table 1 and Supplementary Figures 1A–D). Older adults had slower response times overall on the RMA test than younger adults, but as their switch costs were in the same range as younger adults, this may reflect a slowing of their motor functions rather than a cognitive slowing (Supplementary Figure 1E).

**Table 1:** Summary of the main findings mentioned in the Results (3).

**LMM: Crossing decisions (Nb)**	**β**	***t*-value**	***p*-value**
**Age group**	**−0.14**	**−0.20**	**0.618**
LMM: Time To Impact	β	*t*-value	*p*-value
Age group * traffic density	56.66	2.01	0.045
iMap		*F-value*	*p-value*
Global switch costs		1.12ex04	0.0046
Local switch costs		5.67ex03	0.0072
Pedestrian presence		5.52ex03	0.0081
Local switch cost * Age group		1.35ex03	0.0232
Yuen's test results	*Cohen's d*	*t*-value	*p-value*
Age group (MoCA score)	0.02	0.11	0.985
Age group (zoo map score)	0.05	0.07	0.928
Age group (local switch cost)	0.1	0.003	0.062
Age group (global switch cost)	0.54	0.18	0.062

*The first row is the LMM output for the main effect of age group. The second row is the LMM output for the interaction between age group and traffic density. The next three rows show the mean iMap4 output inside the significant clusters (**Figure 3**). The last four rows show the Yuen's t-test output for the executive function measures compared between age groups. The remaining LMM outputs can be found in the Supplementary Materials*.

### Number of Crossing Decisions

We found no statistically significant difference in the number of crossing decisions between older and younger adults (β = −0.14, SE = 0.28, *t* = −0.20, *p* = 0.618, Table 1 and Supplementary Figure 3). Moreover, a Bayesian LMM, calculated using the blme package in R (Chung et al., 2013), did not indicate any support for a difference in the number of crossing decisions between older and younger adults. We found no other significant effects of our other fixed effects and interactions (Supplementary Table 2).

### Time to Impact Results

**Figure 2:**
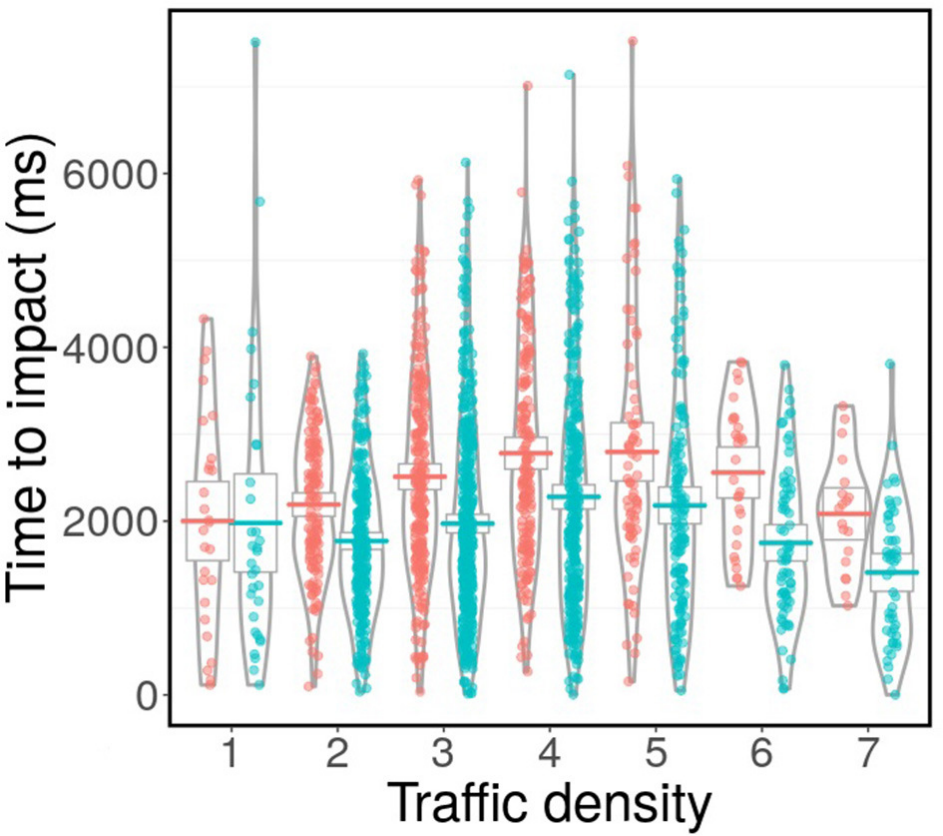
The TTI results for the interaction between age group and traffic density. Red points indicate older adults and blue points indicate younger adults. The plot was created using a combination of the ggplot2 and ggpirate packages in R (Wickham, 2016; Braginsky, 2021).

We found a significant interaction between age and traffic density on TTI (β = 56.66, SE = 28.18, *t* = 2.01, *p* = 0.045, Table 1). Older adults increased their time to impact by more than younger adults when traffic density increased (Figure 2) up until six cars when both groups decreased their time to impact (Figure 2). We found no significant difference between older and younger adults on time to impact (Supplementary Figure 4 and Supplementary Table 1). A Bayesian LMM did not indicate any support for H1.

### IMap4 Results

**Figure 3:**
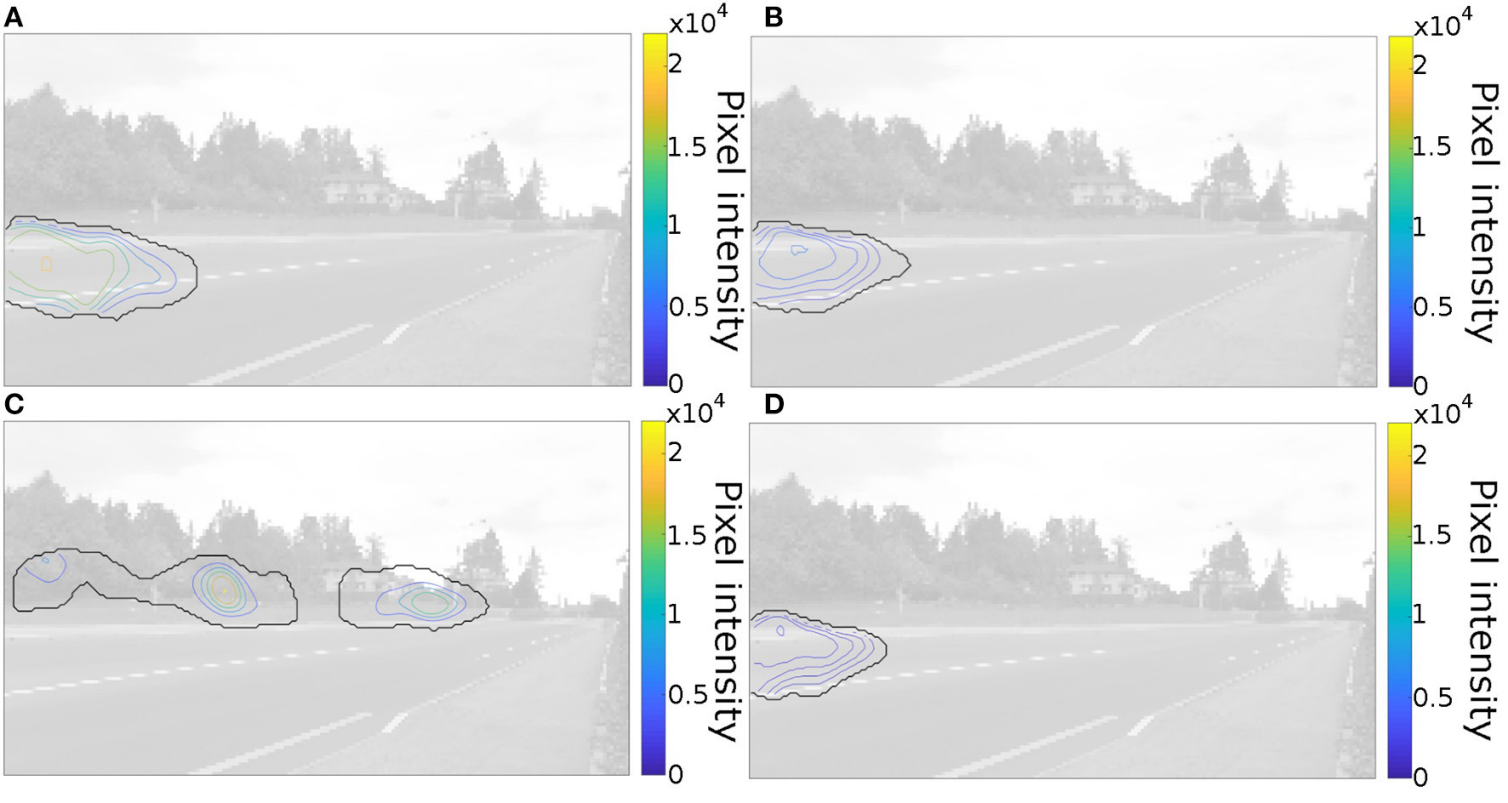
Statistical gaze maps created using iMap4 (Lao et al., 2017) for the main effects of global **(A)** and local **(B)** switch costs on the RMA task. As well as the main effect of pedestrian presence **(C)**, and the interaction between local switch cost and age group **(D)**. Black lines encircle areas gazed at significantly often.

To determine differences in information sampling we computed statistical gaze maps using iMap4 (Lao et al., 2017). We found a main effect of the presence of pedestrian distractors. When the trials contained pedestrian distractors, the participants looked more at areas around the sidewalk where pedestrians are typically found in the videos (Figure 3C and Table 1). We found a main effect of global and local switch costs (Figures 3A,B, respectively, Table 1). Participants with high switch-cost scores looked at the area corresponding to the later part of the vehicles' trajectory, compared to participants with low switch-costs who looked earlier in the trajectory. We also found an interaction between local switch cost and age group (Figure 3D and Table 1). The effect of local switch cost was more pronounced for older adults than for younger adults.

## Discussion

In this study, we aimed to determine whether aging with maintained EF abilities allowed older adults to perform day-to-day tasks such as road crossing to the same level as younger adults. To examine this, we recorded eye movements of young adults and older adults while they watched videos of road traffic and were asked to decide when they believed they could cross the road.

We found that older adults with maintained EFs made a similar number of crossing decisions and left as much time to impact as younger adults, i.e., older adults and younger adults chose similar crossing gaps between vehicles. However, the effect of traffic density on crossing gaps was larger for older adults than for younger adults. Both age groups kept larger crossing gaps (time-to-impact) when traffic density was higher, until the density was too great for large crossing gaps and so the time to impact decreased. This pattern was more pronounced for older adults. This finding suggests that even if older adults with maintained EFs show similar overall crossing behavior, they use different strategies to handle the environmental demands.

We also found that older adults with maintained EFs have similar visual sampling strategies to younger adults. In contrast, adults with large attentional switch costs (local and global) looked more at the area of the road directly in front of them. As these adults are less able than adults with smaller switch costs to switch their attention between targets, they may have fixated the cars further down the road instead of switching their attention to each new vehicle entering the road. Critically, this effect of switch-cost on visual sampling was more marked for older than younger adults (interaction between age and switch costs), again suggesting that despite similar performance levels between the age groups, older adults with maintained EFs use different strategies to perform at the same level as younger adults.

Overall, we found that older adults with maintained EFs make a similar number of crossing decisions, leave the same safety distance between themselves and vehicles when crossing, and show similar information sampling to younger adults. This suggests older adults with maintained EFs can perform day-to-day tasks, such as crossing a road, to a similar level to younger adults. However, older adults with maintained EFs seem to handle environmental constraints, such as the need to perform fast attentional shifts, differently to younger adults. This is reflected by the interaction between age group and traffic density on time-to-impact, as well as the interaction between age group and switch costs on the visual sampling.

Thus, although the overall performance of older adults with maintained EFs is similar to younger adults, the ability to switch attention between relevant targets (as measured by switch-cost or impacted by traffic density) seems to determine, in interaction with age group, how the task is performed.

Nonetheless, the increased crossing gaps for older participants in high traffic density situations, and the fixations later in the trajectory for older participants with high switch costs may lead to a decrease in performance when the situation is more complex. Indeed, it is important to keep in mind that the current study included only one traffic direction. Previous research on crossing behavior in older adults has shown that older adults have more difficulties when cars travel from both directions than one direction (Oxley et al., 1997, 2005). The situation is likely to be more taxing for attentional resources when cars travel from both directions, as participants need to know the location of the vehicles in both visual hemifields and so must switch their attention regularly. Future studies will use more complex situations, including different traffic directions, to investigate whether older adults have difficulties in more complex situations.

It remains undetermined whether, in the present study, participants' overt attention was automatically attracted toward the pedestrian distractors, or conversely whether they voluntarily gazed at pedestrian distractors and covertly attended to cars. This question could be addressed using the approach based on eye-tracking and Steady State Visual Evoked Potentials that we recently developed, and which allowed us to demonstrate that covert shifts of attention reduce visual processing of objects even when they are directly tracked with the eyes (de Lissa et al., 2020).

### Limitations

Although the findings of the present study are robust, their generalizability could be improved in future studies. Indeed, our older participants displayed executive functioning scores in a similar range to that of younger adults. It would be interesting to recruit samples of older participants that reflect the full range of trajectories in cognitive decline. For instance, adding to the experimental design older and younger adults with declining executive functions could help to disentangle the relative contributions of aging and a decline in executive functions. It is important to note that the methods vary substantially across studies in the literature, therefore, it is not entirely surprising that some findings might differ. For instance, in contrast to the present study, in Zito et al. (2015), participants stood in a driving simulator with an immersive 120° horizontal field of view in which the driver mock-up was removed, approaching cars were animations, participants wore a head mounted eye tracker, they could and did move their head, finally they had to take one step forward to indicate they considered it safe to cross the road (instead of a button press).

Overall, we found that older adults with maintained EFs make a similar number of crossing decisions, leave the same safety distance between themselves and vehicles when crossing, and show similar information sampling to younger adults. This suggests older adults with maintained EFs can perform day-to-day tasks, such as crossing a road, to a similar level as younger adults. As these participants are particularly active and many traveled to take part in the experiment *via* walking, public transport, or car, and many lived in the city they may have more experience of traffic than other older adults. Therefore, they may perform at a higher level than the general older adult population due to greater experience. In the future we will address this directly by giving participants a questionnaire to assess their everyday experience of traffic.

We found that older adults made similar crossing decisions to younger adults, despite older adults in the general population typically showing slower walking speeds to younger adults. This is an interesting finding that might be explained by the sample of older participants in this study being physically active in their day-to-day lives. Thus, their walking speeds may be faster than the walking speed of typically aging older adults. In future studies we will measure walking speeds while participants perform real road crossings to determine whether older adults with maintained executive functions are taking into account their walking speeds when making crossing decisions.

The task employed in this study is more complex and realistic than a large number of more reductionist tasks in the literature. Nonetheless, it is important to acknowledge that our task does not reflect the full complexity of real-world situations. For instance, our task included only one traffic direction, did not include a large field of view, head movements, locomotion, and the risks associated with road crossing. Finally, the 2D videos provide only partial distance cues. In future studies, we plan to make the task more immersive by using a larger field-of-view for lab experiments, and more complex by using multiple traffic lanes and directions. We could then corroborate our lab findings using mobile eye-tracking when the participants are standing next to real roads and making real road crossing decisions.

## Conclusion

Overall, our results show that older adults with maintained EFs sample the visual environment similarly and make similar road crossing decisions as younger adults. Thus, this study shows that aging on its own is not necessarily associated with an impairment in a day-to-day task such as road crossing, at least when the task is relatively simple.

Our findings also revealed that both environmental constraints and EF interact with aging in how the road-crossing task is performed. Situations that create high cognitive and perceptual loads combine with aging to require participants to adopt larger and safer crossing gaps. These conservative decisions might indicate strategies aiming at maintaining safe behaviors when the situation is challenging. Indeed, our older adults, despite maintained EFs, probably show an overall decline in perceptual, cognitive, or motor abilities. This is corroborated by a general slowing down indicated by response times on the RMA task. In future studies, more in-depth assessments of other dimensions of aging, such as motor control, and perceptual speed could be used to determine whether these dimensions are the cause of the more cautious strategies used in challenging situations.

Yet, the effects observed on overt attention suggest potential limits of these compensatory strategies. High-switch costs combine with aging to alter visual sampling and reduce fast attention switches toward initial vehicle movements. Thus, older individuals with high switch costs could potentially have difficulties to perform early scanning of vehicles and anticipate their trajectories, making their decisions riskier, particularly when the cognitive and perceptual loads are high. Future studies will assess if situations that are more taxing than the one used in this study lead the compensatory strategies to failure.

Nonetheless, our findings suggest that older pedestrians' safety can be improved through a limitation of the perceptual complexity of the road crossing environment, and a preservation of older adults' EF abilities, potentially through training, exercise, and socialization.

## Data Availability Statement

The datasets presented in this study can be found in online repositories. The names of the repository/repositories and accession number(s) can be found in the article/Supplementary Material.

## Ethics Statement

The studies involving human participants were reviewed and approved by Department of Psychology, Bournemouth University. The patients/participants provided their written informed consent to participate in this study.

## Author Contributions

VN and SM developed the study concept and design. Testing, data collection, and analysis were performed by VN under the supervision of SM, AM, and JW. VN and SM wrote the manuscript. AM and JW provided critical revisions. All authors approved the final version of the manuscript for submission.

## Funding

The project was funded by Bournemouth University.

## Conflict of Interest

The authors declare that the research was conducted in the absence of any commercial or financial relationships that could be construed as a potential conflict of interest.

## Publisher's Note

All claims expressed in this article are solely those of the authors and do not necessarily represent those of their affiliated organizations, or those of the publisher, the editors and the reviewers. Any product that may be evaluated in this article, or claim that may be made by its manufacturer, is not guaranteed or endorsed by the publisher.
