# Supplementary material for: The Relative Contribution of Executive Functions and Aging on Attentional Control During Road Crossing
Source: Front Psychol. 2022 May 12;13:912446. doi: 10.3389/fpsyg.2022.912446 (PMC9133663; doi:10.3389/fpsyg.2022.912446)
Supplement: Supplementary file 1 [file Data_Sheet_1.pdf]

Supplementary Material

1 Supplementary Figures and Tables

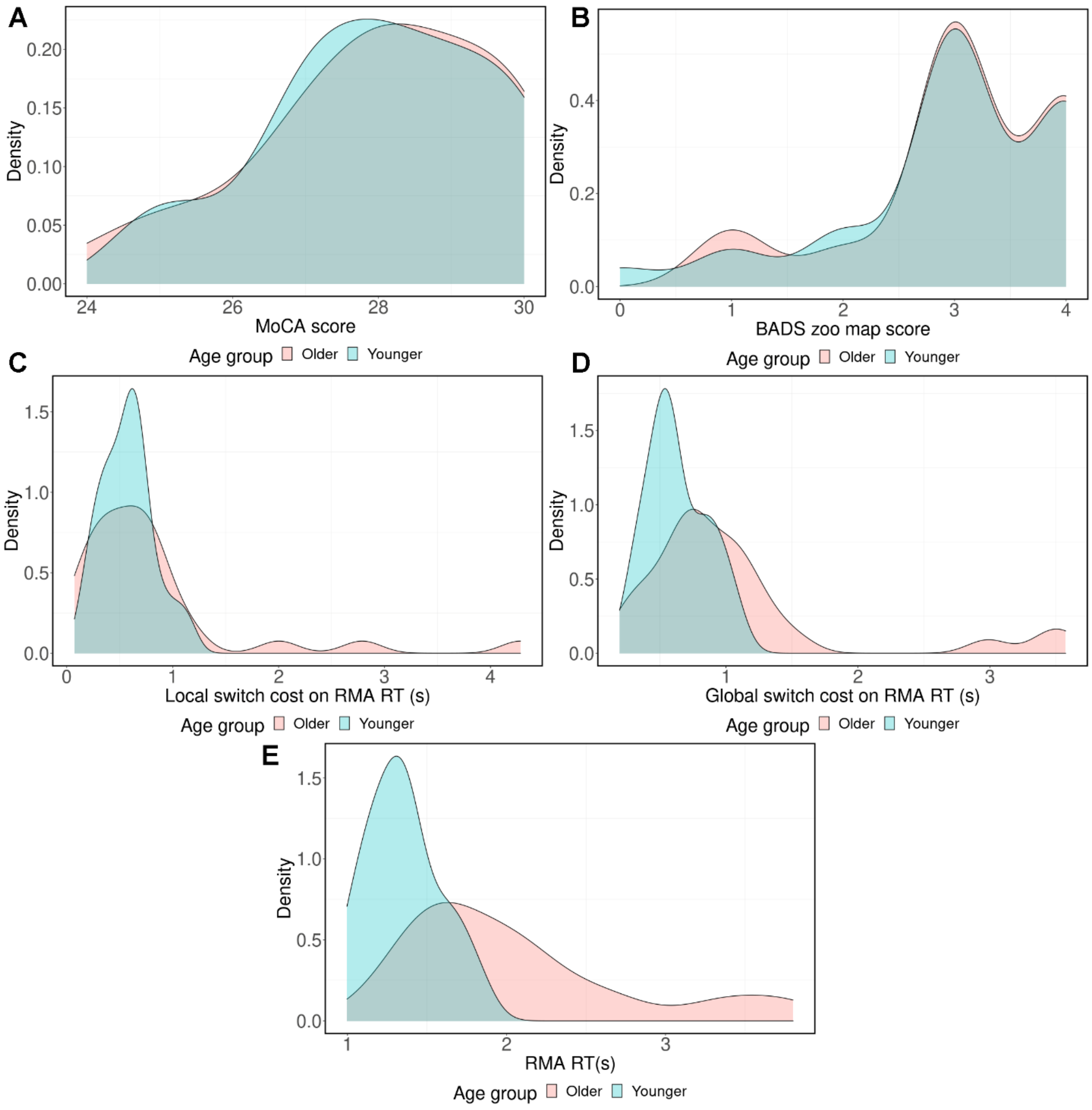

Supplementary Figure 1: EF test results. (A) MOCA, (B) BADS zoo map test, (C) Local switch cost on the RMA task, (D) Global switch cost on the RMA task, (E) median RTs on the RMA task across all blocks. These plots were created using the ggplot2 package in R (Wickham, 2016).

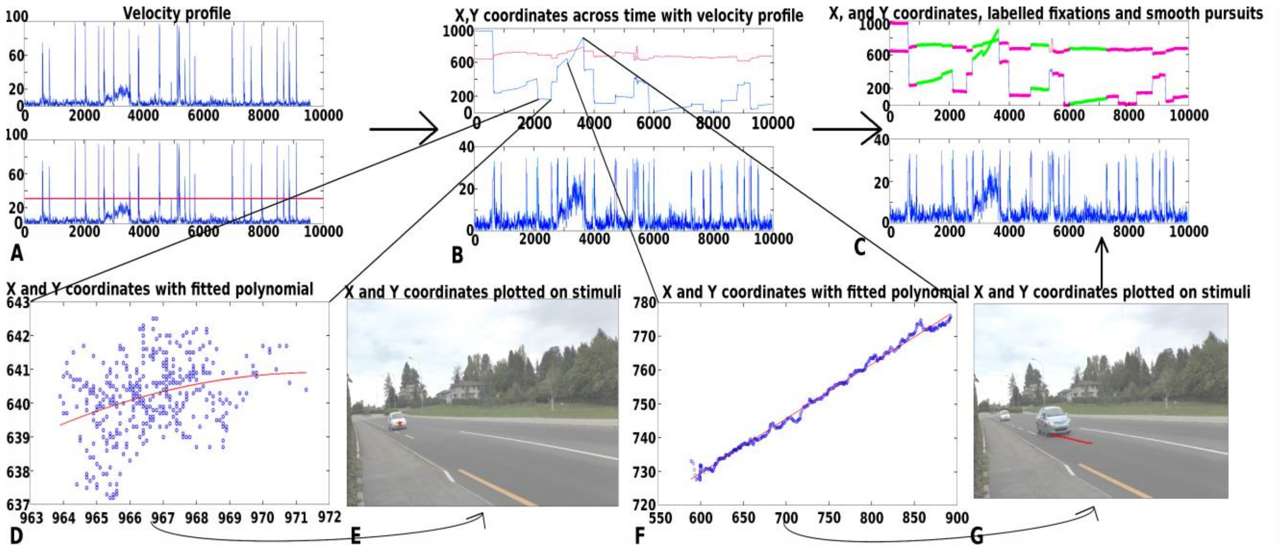

Supplementary Figure 2: Illustration of eye movement parser algorithm. A – velocity threshold to extract saccades (bottom panel). Velocity of eye movement samples (top panel). B – plotting X and Y coordinates of eye movement samples across the whole trial (top panel). D,F – extraction of segments of eye movement samples maintaining a velocity of 30 deg/s for at least 100 ms with a polynomial fitted to the segments. B,G – X and Y coordinates of the segments plotted on matching frames of the experiment stimuli. C – Top panel: completed labelling of eye movements as fixations (pink lines), smooth pursuits (green lines), and saccades (blue lines) for a whole trial.

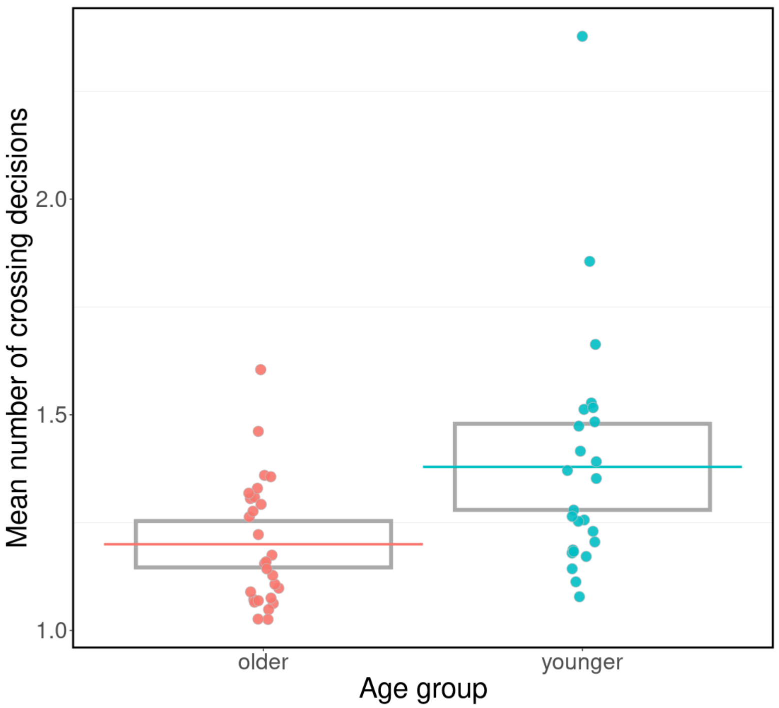

Supplementary Figure 3: Mean number of crossing decisions made by older and younger adults. This plot was created using a combination of the ggplot2 and ggpirate packages in R (Wickham, 2016; Braginsky, 2021).

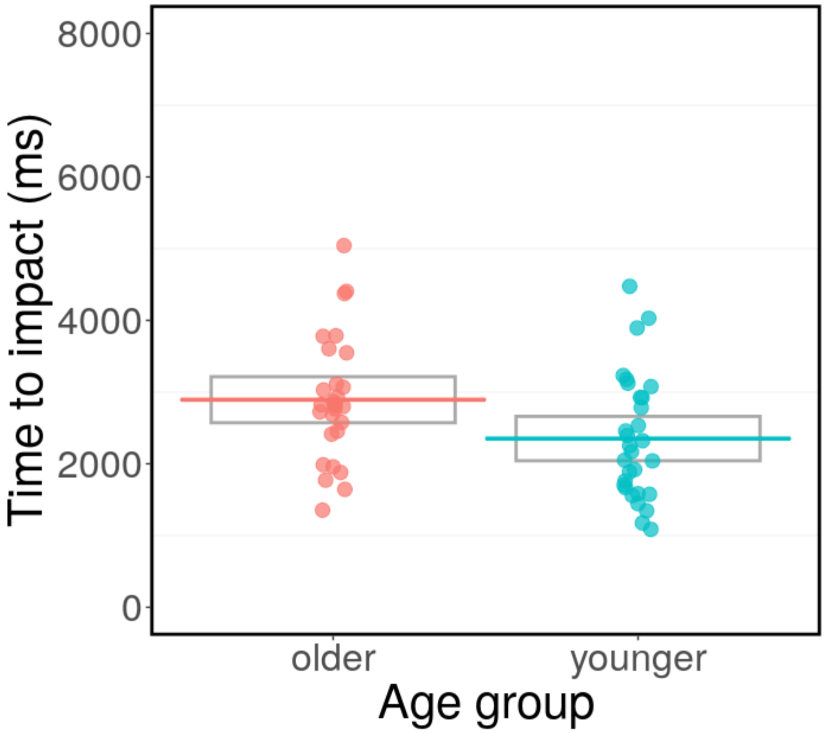

Figure 4 Time to impact for older and younger adults. This plot was created using a combination of the ggplot2 and ggpirate packages in R (Wickham, 2016; Braginsky, 2021).

Table 1. LMM results for TTI. Significant results are highlighted in green.

	Beta	SE	t	P value
Age	-39.48	1069.00	-0.037	0.971
Traffic	65.41	69.44	0.94	0.347
Pedestrians	-149.44	199.89	-0.75	0.455
BADS score	-236.20	199.95	-1.18	0.243
Local switch cost	-275.34	540.74	-0.51	0.614
Global switch cost	-406.27	718.82	-0.57	0.575
Age * traffic	56.66	28.18	2.01	0.045
Age * pedestrians	-60.45	76.25	-0.79	0.428
Age * BADS	352.02	323.76	1.09	0.283
BADS * traffic	-11.66	14.18	-0.82	0.411
BADS * pedestrians	-6.51	39.48	-0.17	0.869
Age * local switch cost	152.18	780.57	0.20	0.846
Local * traffic	57.56	32.02	1.80	0.072
Local * Pedestrians	-77.18	90.72	-0.85	0.395
Age * global switch cost	933.13	993.65	0.94	0.353
Global * traffic	-18.57	41.88	-0.44	0.658
Global * pedestrians	124.39	115.69	1.08	0.282

Table 2. LMM results on the number of crossing decisions.

	Beta	SE	t	p
Age	-0.14	0.28	-0.20	0.618
Traffic	-0.19	0.03	-0.66	0.511
Pedestrian	2.10E-04	0.09	0.002	0.998
BADS	-0.04	0.07	-0.56	0.58
Local Switch Cost	-0.23	0.15	-1.55	0.127
Global Switch Cost	0.15	0.21	0.73	0.467
Age * Traffic	-0.01	0.01	-1.27	0.206
Age * Pedestrian	-0.05	0.04	-1.46	0.146
Age * BADS	3.05E-03	0.08	0.04	0.971
BADS * Traffic	8.23E-03	6.81E-03	1.21	0.227
BADS * Pedestrian	0.02	0.02	0.914	0.361
Age * Local Switch Cost	0.21	0.16	1.29	0.204
Local * Traffic	-3.67E-03	0.01	-0.35	0.729
Local * Pedestrian	-0.02	0.03	-0.67	0.503
Age * Global switch cost	-0.30	0.23	-1.32	0.193
Global * Traffic	0.01	0.02	0.80	0.424
Global * Pedestrian	0.05	0.05	1.15	0.251
